# Advantages of Increasing Histidine to Lysine Ratios on Growth Performance, Blood Parameters and Histidine-Containing Dipeptides for Weaning Piglets

**DOI:** 10.3390/ani16111573

**Published:** 2026-05-22

**Authors:** Diana Siebert, Katharina Schuh-von Graevenitz, Georg Dusel

**Affiliations:** 1 CJ Europe GmbH, 60549 Frankfurt am Main, Germany; 2 Department 1—Agriculture, University of Applied Sciences Bingen, 55411 Bingen am Rhein, Germany; k.schuh-von-graevenitz@ifr-bingen.de (K.S.-v.G.); g.dusel@th-bingen.de (G.D.)

**Keywords:** histidine, SID amino acids, recommendations, weaned piglets, carnosine, growth performance, hematology, anemia

## Abstract

A total of 192 weaned piglets (Topigs TN70), 26 days of age, with an initial body weight of 7.2 ± 0.5 kg, were used to determine the standardized ileal digestible (SID) histidine (His) to lysine (Lys) ratio required to maximize growth performance. Six dietary treatments with graded SID His to Lys ratios ranging from 0.2 to 0.38 were generated with the supplementation of L-His to a grain-based basal diet deficient in His. At the end of the trial (day 41 post weaning), 72 male piglets were euthanized for the analysis of histidine-containing dipeptides, particularly carnosine, in the *M. longissimus dorsi*. In addition, blood samples were collected from 72 piglets to determine plasma urea-nitrogen and hematological parameters. Piglets fed low dietary His (SID His to Lys < 0.27) exhibited hemoglobin concentrations below the reference value, indicating anemia. Muscle carnosine content increased linearly with increasing dietary His and did not reach a plateau within the tested range. Based on growth performance responses, the SID His to Lys ratio required to achieve maximum growth performance in fast-growing piglets is 0.34. These findings emphasize the importance of supervising the SID His to Lys ratio, particularly in practical piglet feed formulations.

## Simple Summary

Histidine is an essential amino acid in swine. Beyond its key role in protein synthesis and growth, it also contributes to numerous metabolic pathways. Insufficient dietary histidine may constrain growth performance in piglets. However, the optimal histidine to lysine ratio for weaned piglets remains poorly defined. Therefore, this study aimed to identify the optimal histidine to lysine ratio for maximizing growth performance in weaned piglets while also exploring its effects on histidine-containing dipeptides (e.g., carnosine) and selected blood parameters. Supplementation of L-histidine to a histidine-deficient basal diet increased growth performance in piglets. An undersupply of dietary histidine was associated with mild anemia, reflected by a lower hemoglobin concentration. Due to animal welfare concerns in connection with the observed anemia, the SID His to Lys ratio should be closely monitored in practical feed formulations. Muscle carnosine displayed a linear increase with increasing dietary histidine. Overall, growth performance and blood parameters suggest an optimal histidine to lysine ratio of 0.34.

## 1. Introduction

The reduction in dietary crude protein (CP) is an effective strategy to improve the profitability and sustainability of swine production [1,2,3]. The availability of crystalline amino acids (AAs) allows for a reduction in CP content without compromising growth performance, provided that the dietary AA supply is aligned with the piglet requirements [1]. Piglet diets are routinely supplemented with lysine (Lys), methionine (Met), threonine (Thr), tryptophane (Trp) and valine (Val). There is growing evidence of the isoleucine (Ile) requirement in piglets [4,5], and Ile is already included in some feeding programs depending on the economic situation and the specific feeding program [1]. If the CP content is reduced further (e.g., below 15%), histidine (His), leucine (Leu), phenylalanine (Phe), and tyrosine (Tyr) may become limiting; however, in practice, these are often not considered in feed formulations for piglets.

Histidine is an essential proteogenic amino acid in pigs and is consequently required for protein synthesis and overall growth. His may become limited when dietary CP is reduced, but also when diets are formulated with specific raw materials that have a low His content. In particular, rye (SID His 1.7 g/kg) and barley (SID His 1.8 g/kg) contain a relatively low digestible His content [6] and diets based on these cereals may therefore be at greater risk of His limitation. In contrast, typical diets based on corn (SID His 1.9 g/kg) and soybean meal (CP > 45%; SID His 11.7 g/kg) [6] are more likely to provide sufficient digestible His.

A prerequisite for an accurate formulation is the knowledge about the animals’ amino acid requirements. The NRC recommends a His to Lys ratio of ~0.34 [7]. However, this recommendation is largely based on a factorial approach to estimate growth requirements and, in the case of His, relied solely on a single empirical study [8]. Since this study was conducted more than three decades ago, its applicability to modern piglet genotypes may be limited given substantial genetic progress. More recent recommendation systems suggest lower His to Lys ratio of approximately 0.29 to 0.31 for weaned piglets [9], which is in line with the current Dutch recommendations of 0.30 [10]. The relatively large variations between the recommendations indicate that research on His requirements in piglets remains scarce and that His requirements have still not been precisely defined.

Histidine has a wide variety of functions and is also a component of specific body proteins [11], which needs to be taken into account when estimating dietary requirements. In particular, His is an important component of hemoglobin (Hb), the oxygen-transport protein in red blood cells. Consequently, an inadequate His supply may affect Hb synthesis. In a rat model, hematocrit (Htk) and hemoglobin concentrations declined over time during His depletion [12]. Under practical rearing conditions, anemia in piglets—indicated by low Hb—has been associated with reduced growth performance [13]. Although low Hb in piglets is predominantly caused by iron deficiency [13], it remains unclear whether insufficient dietary His can also cause a practically relevant reduction in Hb concentration in piglets.

Beyond its role in Hb synthesis, His can be metabolized together with ß-alanine to form the dipeptide carnosine. Carnosine and its methylated analogues, anserine and ophidine, are collectively referred to as histidine-containing dipeptides (HCDs). A notable concentration of HCDs is found mainly in skeletal muscle and, to a lesser extent, in the brain [14]. A variety of physiological functions are served by HCDs with protection against oxidative stress considered one of the most important [15]. The imidazole ring delivered from His can chemically interact with reactive oxygen species, which is thought to underpin the antioxidant activities of His and HCDs [16]. In vitro, carnosine protects against oxidative DNA damage and experimentally induced oxidation of liposomes [17]. Furthermore, HCDs act as an intracellular proton buffer, thereby contributing to the maintenance of muscle acid-base balance [14].

Histidine can also be metabolized to histamine having an important role in digestion, particularly by stimulating gastric acid secretion [18]. In addition, histamine appears to be involved in immune regulation through the activation of histamine-receptors [19]. Recent research in broilers further suggests an effect of His on the immune system [20]. In a His dose–response study, the authors applied a microbial challenge consisting of an in-feed coccidiosis challenge followed by an oral bacterial challenge. Challenged birds required higher levels of standardized ileal digestible (SID) His to achieve performance comparable to unchallenged birds [20], which may be related to increased utilization of His and its metabolites during immune activation.

Dietary His exerts a variety of functional effects and is therefore not only relevant for growth performance. The aim of this trial was to determine the optimal SID His to Lys ratio for nursery pigs and to evaluate the effect of increasing SID His to Lys ratios on functional parameters, including HCD concentrations in muscle and selected blood parameters.

## 2. Materials and Methods

### 2.1. Animals and Housing

A feeding trial was conducted using 192 (26 days old) weaned piglets (TOPIGS-SNW GmbH, 48308 Senden, Germany, TN 70, females and castrated males equally distributed). At the start of the trial, piglets were individually weighed (7.2 kg ± 0.5 kg) and fitted with individual ear tags. Piglets were housed in groups of four per experimental pen (sex-balanced, 1 m^2^; slatted floor) equipped with a nipple drinker and an automatic feeder. Pens (eight replicate pens per treatment) were randomly allocated to one of six dietary treatments, with treatments evenly distributed across the facility. The trial lasted 41 days and was divided into two feeding phases (Phase 1: 0–14 days; Phase 2: 15–41 days), to reflect the changing lysine and energy requirements of piglets during the time periods accordingly. Feed and water were provided ad libitum. Feed intake was recorded per pen. Mortality was monitored daily throughout the rearing period (0–41 days). Cotton ropes (10 mm diameter) and plastic balls were provided in each pen as enrichment material.

### 2.2. Experimental Diets

A basal diet based on wheat, barley, corn, rye and soybean meal, without supplemental amino acids was manufactured at a commercial feed mill (Mischfutter Werke Mannheim GmbH, 68169 Mannheim, Germany). A representative sample of the basal diet was analyzed for its AA content prior to the trial. Based on the analytical results, crystalline-free AAs were added at the Research Feed Unit of the University of Applied Science Bingen (55411 Bingen, Germany) to produce a diet that met the piglets AA requirements (except for His) (Table 1). To allow piglet performance to be related specifically to the His to Lys ratios, the digestible Lys content of the experimental diets was reduced by approximately 12% compared with CJ Europe GmbH recommendations, to 1.10% in phase 1 and 1.05% in phase 2. Sub-batches of the trial diets were supplemented with graded levels of L-His (BestAmino L-Histidine HCl·H_2_O, 72%, CJ Bio Seoul, Korea) prior to pelleting, resulting in SID His to Lys ratios of 0.20, 0.24, 0.27, 0.31, 0.35 and 0.38, respectively. Digestible coefficients of the raw materials were taken from published tables [6].

### 2.3. Diet Analysis

All phase 1 and phase 2 diets were analyzed for proximate composition and AA content according to the official methods of the VDLUFA [21]. The analyzed nutrient concentrations are presented in Table 2 and Table 3, respectively. Based on the analyzed (total) values the His to Lys ratio ranged from 0.27 to 0.38 in phase 1 and from 0.25 to 0.38 in phase 2. Considering the typical measurement uncertainty in amino acid analytics and noticing that the total His amount is consecutively increasing from treatment 1 to 6 the calculated and analyzed values were in good agreement.

### 2.4. Performance Measurments, Blood and Tissue Sampling, Deterimination of Blood Parameters and Histidine-Containing Dipeptides in Muscle

Piglets were weighed individually on a weekly basis. Feed residues were measured at the same intervals. The amount of feed offered was continuously recorded each time the feeder was refilled. Animals were observed twice daily by the research site animal supervisor and experienced and trained personnel; any deviations in appearance or behavior were documented in the farm logbook. Animals in poor condition were monitored more frequently. Mortality, including presumed cause of death when available, was recorded.

Average daily body weight gain per piglet (BWG), average daily feed intake per piglet (FI) and feed conversion ratio corrected for mortality (FCR) were calculated for phase 1 (days 0–14), phase 2 (days 15–41), and the overall trial period (days 0–41), respectively.

As part of routine veterinary health control, 12 piglets per treatment were randomly selected for blood sampling on day 40. Blood samples were allowed to clot for 30 min at room temperature and were then centrifuged for 10 min at 2000× *g*. After centrifugation, the resulting serum samples were immediately sent to an external laboratory (LABOKLIN GmbH & Co. KG, Bad Kissingen, Germany) for analysis of plasma urea-nitrogen (PUN) and hematological parameters (hematocrit, hemoglobin, leucocytes, erythrocytes and thrombocytes, respectively).

At the end of the trial (d 41), 12 male piglets per treatment with body weights closest to the treatment mean were selected and slaughtered. A muscle sample (about 200 g) was collected from the *M. longissimus dorsi* and immediately cooled. After collection, samples were transported to the Laboratory for Animal Nutrition and Animal Product Quality (Lanupro) at Ghent University’s Department of Animal Sciences and Aquatic Ecology for determination of HCDs (carnosine and anserine). The carnosine and anserine content were determined using the method described by Barbaresi et al. [22].

### 2.5. Statistical Analysis

Data were checked for normality and homogeneity of variances prior to the statistical analysis. Outliers were assessed, no extreme values were identified and therefore no data were excluded. Study outcomes were analyzed by one-way analysis of variance (ANOVA) using IBM SPSS Statistics (Version 29). For body weight (BW) and BWG, treatment and sex were included as fixed factors, with individual piglets as experimental units and initial BW included as a covariate. For FI and FCR, the pen served as the experimental unit. For muscle and blood parameters, the individual pig was considered the experimental unit and treatment was included as a fixed factor. In the case of significant differences between the treatment groups (*p* ≤ 0.05), means were compared using a post hoc test (Tukey HSD-Test).

Performance responses indicated a quadratic dose–response relationship. Accordingly, a quadratic model was fitted to the data, and the SID His to Lys ratio required to achieve maximal (vertex) performance was estimated.

$$\mathrm{The}\;\mathrm{quadratic}\;\mathrm{model}:\;y=a+\left(b_{1}\cdot x\right)+\left(b_{2}\cdot x^{2}\right)$$

where “y” represents the measured response variable, “x” denotes the dietary AA level, “a” is the intercept, “b_1_” describes the linear component and “b_2_” the curvature of the response. The observed performance data did not support the application of a broken-line or exponential model.

## 3. Results

### 3.1. Losses and Mortality

The trial ran without complications, and no medical treatment was necessary. Consequently, overall mortality was very low. Over the entire trial period, one animal was lost due to cardiovascular collapse (treatment 3), and two animals were excluded from the trial because of very poor growth (treatment 3 and treatment 4). Treatment had no effect on mortality.

### 3.2. Growth Performance

At start of the trial, animals weighted on average 7.2 ± 0.5 kg, with no statistically significant differences among treatments. At the end of the study, all His supplemented treatments resulted in higher BW than the His-deficient basal diet. The highest final BW was observed in the His-supplemented treatment with a SID His to Lys ratio of 0.31 (treatment 4), showing an approximately 14% (3 kg) increase in BW compared with the basal diet (Table 4). Numerically, BWG increased with increasing His supplementation and reached a maximum at a SID His to Lys ratio of 0.31. With higher His additions (SID His to Lys 0.35 and 0.38, respectively) performance decreased slightly (Table 4), although it remained significantly higher than the basal diet (SID His to Lys 0.20). A comparable quadratic response pattern was also visible for the FI and the FCR (Table 5).

### 3.3. Estimation of SID His to Lys Requirement in Piglets

Performance and blood parameters showed a quadratic response. Table 6 presents the calculated vertex of SID His to Lys ratios along with the corresponding SID His percentage in the diet required to maximize performance. The estimated SID His to Lys ratio was lowest for Urea-N (SID His to Lys: 0.32) and highest for FI and FCR (SID His to Lys: 0.34). Estimates for final BW, hematocrit and hemoglobin were intermediate (SID His to Lys: 0.33). Estimation of the recommended SID His to Lys for BWG as well as the performance parameters at feeding phase 1, was not possible.

### 3.4. Blood Parameters

Urea-N, Htk and Hb showed a quadratic response, with an optimum at a SID His to Lys ratio of 0.27 (Table 7). In treatment 1 and 2, the measured Hb values were below the laboratory reference range; therefore, the animals can be classified as anemic. Erythrocytes, leukocytes, and thrombocyte counts showed high variability, and no statistically significant differences were observed among treatments.

### 3.5. Histidine-Contaning Dipeptides

Increasing His content in the feed led to a significantly higher carnosine concentration in the *M. longissimus dorsi*. The lowest carnosine concentration (70.0 mg/100 g of tissue) was observed at a SID His to Lys ratio of 0.20 and increased linearly up to a maximum of 383.4 mg/100 g of tissue at a SID His to Lys of 0.38. In contrast, anserine concentration showed some variation but remained overall at a comparable low level (Table 8).

## 4. Discussion

In the present study, the optimal standardized ileal digestible (SID) His to Lys ratio was estimated at 0.33 for final BW and at 0.34 for FI (days 0–41), and FCR (days 15–41), respectively. These estimates and the quadratic response in growth performance closely aligned with the quadratic response in PUN, which was minimized at a SID His to Lys of 0.32. Urea is the principal end-product of amino acid catabolism and ammonia detoxification in the liver. Because PUN concentration is closely linked to hepatic urea synthesis and urinary N excretion, it is commonly used as an indicator of nitrogen utilization efficiency, or at least as a proxy for urinary N excretion in pigs [23,24]. Accordingly, lower PUN concentration may reflect reduced N losses and a greater partitioning of N toward body protein deposition and whole-body protein synthesis. The fact that both growth performance and PUN were optimized at His to Lys ratio between 0.32 and 0.34 may therefore suggest improved protein synthesis and N utilization within this range. However, because the present experimental design was not intended to qualify N balance of N retention, interpretation of the PUN as a measure of N efficiency should be made with caution.

With respect to growth performance, the present estimates are consistent with the SID His to Lys ratio of 0.34 suggested by NRC (2012) [7]. A dose–response study using cereal–soybean meal-based diets in 10–20 kg piglets also reported an optimal His to Lys ratio of 0.32 based on a curvilinear plateau model [25]. In contrast, two dose–response experiments (7–11 kg) done in the US with corn-soybean meal-based diets concluded that the optimal SID His to Lys ratio for growth performance does not exceed 0.31 [26]. Notably, those experiments used a comparatively high CP level (>18%) and pharmacological doses of zinc oxide (0.25% as fed) [26], which differ substantially from the present study (low CP, ~15% and no zinc oxide). Such differences in the formulation may contribute to discrepancies in estimated requirements. Conversely, a study conducted in Germany suggested an even lower optimal SID His to Lys ratio of approximately 0.28 when using a very low CP diet (~14%) based on wheat and barley [27]. Nevertheless, the authors acknowledged that a His to Lys ratio of 0.30 may be advisable to provide a safety margin in low CP feed formulation [27].

The aforementioned studies [25,26,27] estimated the optimal His to Lys ratio solely based on growth performance, and no information was provided about HCD status of the piglets or other physiological parameters. However, a recent review in poultry summarized that dietary His deficiency may lead to HCD breakdown in skeletal muscles [28], which could partly explain relatively flat performance responses in dose–response trials and thereby interfere with requirement estimation. Similarly, the current study could not estimate an optimal His to Lys ratio in phase 1 due to a weak growth response. One possible explanation is that the analyzed His concentrations in the phase 1 diets were higher than intended, potentially attenuating the effects of His deficiency. In addition, HCD mobilization during phase 1 may have contributed to the lack of response. This interpretation aligns with observations from a recent dose–response study in nursery piglets (7 to 11 kg BW) [29], which broadly corresponds to the phase 1 period in the current trial. In that study, increasing dietary His to Lys ratios did not affect growth performance; therefore, no performance-based optimum could be derived. Nonetheless, the authors reported a quadratic response in muscle carnosine concentrations, with a maximum at a SID His to Lys ratio of 0.38 [29]. In contrast, the present study did not observe a quadratic response for carnosine concentration; instead, carnosine concentration in *M. longissimus dorsi* increased linearly with increasing dietary His to Lys ratio. Consequently, the carnosine data did not reach a plateau, and no breakpoint could be estimated. It should be noted that Cheng et al. (2023) evaluated five SID His to Lys ratios ranging from 0.26 to 0.50 [29], whereas the present study increased supplementation only up to 0.38. The upper level corresponds to the breakpoint reported by Cheng et al. (2023) [29]. Based on the response pattern in the present study, it is plausible that carnosine concentration might have plateaued or decreased at SID His to Lys ratios above 0.38. This is supported by the diminishing incremental response at the highest supplementation levels: carnosine increased from treatment 4 (SID His to Lys 0.31) to 5 (SID His to Lys 0.35) by 45.2%, but only by 5.29% from treatment 5 (SID His to Lys 0.35) to treatment 6 (SID His to Lys 0.38). However, this hypothesis cannot be verified within the design of the present study.

Anserine concentration in *M. longissimus dorsi* showed a slight increase in His supplemented treatments, but no consistent response pattern was evident. A weak or absent anserine response is consistent with a Spanish field study in finishing pigs [30], in which pigs received either a commercial control diet (SID His to Lys 0.37) or a diet supplemented with L-His (SID His to Lys 0.60) during the final 12 weeks before slaughter (~130 kg BW). While carnosine concentration in the *M. longissimus dorsi* increased significantly in the His supplemented group, anserine showed only a slight numerical increase [30]. These observations support the concept that, among HCD, carnosine is the most relevant dipeptide in pigs, whereas anserine may be of lesser physiological importance [14].

Hemoglobin consists of a heme iron and four polypeptide globin chains and contains about 8% His [29,31]. In particular, His residues located at distal positions within the globin chains can contribute to hydrogen ion buffering and may also affect oxygen binding [31]. In rat models, in addition to carnosine, Hb can be degraded to supply His and thereby mitigate the adverse effects of a His-deficient diet [12]. In the present study, Hb and Htk were maximized at a SID His to Lys of 0.33. This is slightly lower than the SID His to Lys of 0.36 reported to optimize Hb in nursery piglets (7 and 11 kg BW) by Cheng et al. (2023) [29]. Using SID His to Lys ratios ranging from 0.26 to 0.50, Cheng et al. (2023) observed a relatively flat quadratic response, with an average Hb concentration of 12 g per dL at the estimated breakpoint [29]. This value agrees with Hb concentrations observed in the present study for treatments with SID His to Lys ratios above 0.27 and falls within the laboratory reference range (10.8–14.8 g per dL) [32]. By contrast, the two lowest SID His to Lys ratios (0.20 and 0.24) resulted in Hb concentrations of 9.8 and 10.1 g per dL, respectively), i.e., below the reference range [32]. Although reduced Hb concentration and anemia in piglets are well described in the context of iron deficiency [13], the possibility that dietary His deficiency may also lead to anemia is less well stablished and warrants further investigation.

In the present study, growth parameters showed a quadratic response pattern. The underlying mechanism for this response is not fully understood. One plausible explanation involves the metabolism of His to histamine. Histamine acts as a neurotransmitter and is involved in appetite regulation [33], and it also contributes to digestive processes by stimulating the secretion of pepsinogen and hydrochloric acid [18] in the chief cells and parietal cells. A dietary His deficiency may impair histamine synthesis and thereby reduce feed intake. In line with this hypothesis, the present study observed a significant reduction in FI; hence, the lowest BWG is at the lowest SID His to Lys ratio (0.20). Similar reductions in FI and BWG with His-deficient diets have been reported previously [25,26,27]. Conversely, excessive histamine may also affect the activity of histaminergic neuronal systems [33]. Activation of H_1_-receptors and/or inhibition of H_3_-receptors have been implicated in satiety regulation and suppression of feed intake [34]. This is supported by evidence from a rat model in which high dietary His levels induced anorexia [35]. In the present study, despite not being statistically significant, FI showed a slight numerical decrease at the highest SID His to Lys ratio (0.38). This observation may suggest that performance could decline at SID His to Lys ratios above 0.38; however, other researchers could not observe a reduction in FI or other performance parameters at SID His to Lys ratios of 0.40 [25], 0.44 [26] or 0.49 [27].

An additional consideration is that His competes with other large neutral amino acids (LNAA; e.g., leucine, isoleucine, valine, tryptophan, threonine and phenylalanine) for transport across the blood–brain barrier via shared carrier systems [36]. Potential LNAA interactions may be more pronounced under dietary conditions such as those in the present trial, namely a low CP level (~15%) combined with sub-limiting SID Lys (1.10% and 1.05%, respectively). While a recent study in finishing broilers found no effect of a high Leu to Lys ratio on the optimal Trp to Lys ratio, suggesting limited practical relevance of LNAA interactions in that model [37], comparable evidence is rare in piglets.

The aim of this study, amongst other objectives, was to determine the SID His to Lys ratio required to optimize growth performance in piglets. Various statistical models can be applied; however, the best fitting model also depends on the characteristics of the data. Herein, the data did not support the use of broken-line or exponential models; consequently, a quadratic model was chosen as the best fitting model. Requirements were estimated at the vertex (maximum) of the response curve to provide the highest possible security for practical feed formulation, where His is often not considered limiting because optimal His to Lys ratios in swine have rarely been investigated.

## 5. Conclusions

Dietary His supply markedly influenced growth performance and selected physiological indicators in piglets. A His-deficient diet significantly impaired performance, and piglets fed SID His to Lys ratios below 0.27 exhibited Hb values below the laboratory reference range, suggesting an increased risk of anemia. These findings highlight the importance of monitoring the SID His to Lys ratio in practical feed formulations, particularly under low CP conditions. Based on the quadratic response of growth performance, a SID His to Lys ratio of 0.34 is recommended for piglets from 7 to 25 kg BW to maximize performance. In addition, carnosine concentration in skeletal muscle increased linearly with increasing dietary His and did not reach a plateau within the range evaluated, indicating that muscle carnosine deposition continued to increase up to the highest inclusion level tested.

## Figures and Tables

**Table 1:** Feed ingredients and chemical composition [g/kg] of the basal test diets as fed in phase 1 (day 0–14) and phase 2 (day 15–41) with a deficient standardized ileal digestibility (SID) histidine (His) to SID lysine (Lys) ratio of 0.2.

Ingredients [% of the Diet]	Phase 1 (d 0–14)	Phase 2 (d 15–41)
Wheat	37.9	40.7
Barley	15.4	28.6
Corn	20.6	10.2
Rye	6.15	8.15
Soybean meal (48% CP)	8.20	6.75
Whey powder	5.15	
Wheat bran	2.55	2.55
Mineral and AA premix ^1^	0.51	0.51
Soy oil	1.55	0.900
Monocalcium phosphate (MCP)	0.51	0.41
Salt	0.41	0.41
Calcium carbonate	1.14	0.82
Calculated ingredients [%]		
Metabolizable energy (ME), MJ/kg	13.6	13.0
Net Energy (NE), MJ/kg	10.7	10.0
Crude protein	15.2	15.0
Crude fat	4.80	3.20
Crude fiber	2.80	3.30
SID Lysine	1.10	1.05
SID Methionine	0.40	0.37
SID Threonine	0.80	0.75
SID Tryptophane	0.25	0.23
SID Valine	0.88	0.82
SID Isoleucine	0.68	0.63
Total calcium	0.700	0.540
Total phosphorus	0.460	0.430

CP stays for crude protein. ^1^ Provides per kg premix: 3,500,000 IU of Vitamin A; 400,000 IU of Vitamin D3; 30,000 mg of Vitamin E; 400 mg of Vitamin K3; 50,000 mcg of Vitamin H (Biotin); 666.67 mg of Folic acid; 700 mg of Vitamin B1; 1400 mg of Vitamin B2; 1200 mg of Vitamin B6; 11,111.11 mcg of Vitamin B12; 7000 mg of Niacinamide (niacin); 4384 mg of Calcium-D-pantothenic acid; 32,904 mg of Betaine-HCl; 111,111.11 mg Choline chloride; 15,000 mg of Fe (sulfate); 15,000 mg of Fe-glycine; 20,750 mg of Cu (Cu-chelate), 10,000 mg of MnO; 15,000 mg of ZnO; 10,000 mg of Zn-glycine; 200 mg of I; 70 mg of Se; and 10 mg of Se-methionine.

**Table 2:** Analyzed total amino acids and proximate composition as fed of phase 1 diets (days 0–14) containing six graded levels of histidine.

Total Amino Acid Content [%]	Treatment 1 (*0.20*) *	Treatment 2 (*0.24*)	Treatment 3 (*0.27*)	Treatment 4 (*0.31*)	Treatment 5 (*0.35*)	Treatment 6 (*0.38*)
Arginine	1.17	1.19	1.11	1.16	1.19	1.18
Histidine	0.30	0.32	0.33	0.37	0.40	0.43
Isoleucine	0.66	0.65	0.68	0.70	0.73	0.71
Leucine	1.26	1.24	1.25	1.30	1.34	1.33
Lysine	1.11	1.10	1.06	1.10	1.11	1.12
Methionine plus Cysteine	0.70	0.72	0.70	0.70	0.73	0.70
Threonine	0.78	0.79	0.77	0.81	0.83	0.81
Phenylalanine	0.52	0.55	0.51	0.51	0.54	0.54
Valine	0.85	0.88	0.89	0.93	0.97	0.95
Proximate [%]						
Crude protein	14.70	15.30	15.10	14.80	15.00	15.30
Crude fat	4.00	3.80	3.90	4.00	3.90	3.80
Crude ash	4.00	4.10	4.20	4.20	3.90	4.40
Crude fiber	2.60	2.40	2.50	2.70	2.70	2.50

* Italic values in brackets refer to the calculated SID His to SID Lys ratio; SID = standardized ileal digestible.

**Table 3:** Analyzed total amino acids and proximate composition as fed of phase 2 diets (day 15–41) containing six graded levels of histidine.

Total Amino Acid Content [%]	Treatment 1 (*0.20*) *	Treatment 2 (*0.24*)	Treatment 3 (*0.27*)	Treatment 4 (*0.31*)	Treatment 5 (*0.35*)	Treatment 6 (*0.38*)
Arginine	1.24	1.26	1.24	1.23	1.26	1.28
Histidine	0.26	0.31	0.32	0.34	0.39	0.42
Isoleucine	0.68	0.70	0.69	0.69	0.69	0.69
Leucine	1.31	1.32	1.30	1.29	1.31	1.29
Lysine	1.04	1.07	1.05	1.03	1.09	1.08
Methionine plus Cysteine	0.89	0.87	0.87	0.87	0.87	0.87
Threonine	0.85	0.80	0.82	0.78	0.81	0.82
Phenylalanine	0.50	0.53	0.49	0.49	0.56	0.52
Valine	0.93	0.93	0.92	0.92	0.95	0.96
Proximate [%]						
Crude protein	14.60	15.10	14.50	14.80	15.30	15.70
Crude fat	2.50	2.60	2.60	2.40	2.60	2.70
Crude ash	3.50	4.30	3.50	3.50	4.20	4.40
Crude fiber	2.70	3.20	2.80	3.00	3.50	3.50

* Italic values in brackets refer to the calculated SID His to SID Lys ratio; SID = standardized ileal digestible.

**Table 4:** Body weight (day 41) and body weight gain in the different feeding phases in piglets fed different His to Lys ratios.

Treatment (*SID His to Lys*)	Body Weight, Day 41 [kg]	Body Weight Gain, Phase 1 Day 0–14 [g/d]	Body Weight Gain, Phase 2 Day 15–41 [g/d]	Body Weight Gain Total Trial Day 0–41 [g/d]
1 (*0.20*)	22.1 ^a^	274	410 ^a^	364 ^a^
2 (*0.24*)	24.5 ^b^	274	500 ^b^	423 ^b^
3 (*0.27*)	24.8 ^b^	266	515 ^b^	431 ^b^
4 (*0.31*)	25.1 ^b^	286	516 ^b^	438 ^b^
5 (*0.35*)	24.5 ^b^	264	504 ^b^	422 ^b^
6 (*0.38*)	24.5 ^b^	272	502 ^b^	424 ^b^
SEM	0.235	4.535	6.633	5.316
*p*-value				
Treatment ^1^	<0.001	0.763	<0.001	<0.001
Regression quadratic ^2^	<0.001	0.979	0.114	0.173

^1^ *p*-value based on ANOVA (GLM) using treatment and sex as fixed factors (incl. interaction; sex was distributed equally in pens), BW0 was used as a covariate; ^a,b^ means with different superscripts differ significantly: *p* < 0.05 (post hoc Tukey HSD); ^2^ *p*-value based on quadratic regression analysis with increasing level of histidine in the diet. Italic values in brackets refer to the calculated SID His to SID Lys ratio; SID = standardized ileal digestible.

**Table 5:** Average daily feed intake and FCR during the different feeding phases in piglets fed different His to Lys ratios.

Treatment (*SID His to Lys*)	Daily Feed Intake Phase 1 Day 0–14 [g/d]	Daily Feed Intake Phase 2 Day 15–41 [g/d]	Daily Feed Intake Total Trial Day 0–41 [g/d]	FCR Phase 1 Day 0–14 [g/g]	FCR Phase 2 Day 15–41 [g/g]	FCR Total Trial Day 0–41 [g/g]
1 (*0.20*)	391	696 ^a^	592 ^a^	1.43	1.70 ^b^	1.63
2 (*0.24*)	385	818 ^b^	670 ^b^	1.41	1.64 ^ab^	1.59
3 (*0.27*)	378	795 ^b^	653 ^b^	1.42	1.64 ^ab^	1.59
4 (*0.31*)	391	812 ^b^	668 ^b^	1.39	1.62 ^a^	1.58
5 (*0.35*)	380	826 ^b^	673 ^b^	1.44	1.64 ^ab^	1.60
6 (*0.38*)	380	816 ^b^	667 ^b^	1.40	1.63 ^ab^	1.58
SEM	5.525	8.882	6.594	0.012	0.008	0.006
*p*-value						
Treatment ^1^	0.975	<0.001	<0.001	0.825	*0.051*	0.119
Regression Quadratic ^2^	0.964	<0.001	0.001	0.720	0.045	0.102

^1^ *p*-value based on ANOVA (GLM) using treatment and sex as fixed factors (incl. interaction; sex was distributed equally in pens), BW0 was used as a covariate; ^a,b^ means with different superscripts differ significantly: *p* < 0.05 (post-hoc Tukey HSD); ^2^ *p*-value based on quadratic regression analysis with increasing level of histidine in the diet. Italic values in brackets refer to the calculated SID His to SID Lys ratio; SID = standardized ileal digestible.

**Table 6:** Estimation for nutritional requirements of histidine (% in the diet) or the standardized ileal digestible (SID) His to Lys ratio to achieve maximum performance.

Parameter	% SID His in the Diet	SID His:Lys
Body weight [kg] d 41	0.36	0.33
Daily feed intake [g/d], d 15–41	0.37	0.34
Daily feed intake [g/d], d 0–41	0.37	0.34
FCR [g/g], d 15–41:	0.37	0.34
Urea-N [mg/dL]	0.36	0.32
Hematocrit [%]	0.36	0.33
Hemoglobin [g/dL]	0.36	0.33

**Table 7:** Effect of increasing histidine (His) supplementation on plasma urea nitrogen (PUN) and small blood count parameters in piglets after 6 weeks of being fed with graded levels of His.

Treatment (*SID His to Lys*)	PUN [mg/dL]	Erythrocytes [M/µL]	Hematocrit [%]	Hemoglobin [g/dL]	Leukocytes [K/µL]	Thrombocytes [K/µL]
1 (*0.20*)	9.4 ^b^	6.8	35.5 ^a^	9.8 ^a^	15.4	536.2
2 (*0.24*)	7.5 ^ab^	6.5	35.8 ^a^	10.1 ^ab^	16.4	495.7
3 (*0.27*)	6.6 ^a^	7.1	41.1 ^b^	11.7 ^c^	18.6	521.8
4 (*0.31*)	6.7 ^a^	6.9	39.7 ^ab^	11.6 ^c^	17.7	471.9
5 (*0.35*)	7.1 ^ab^	6.8	38.6 ^ab^	11.2 ^bc^	18.3	449.2
6 (*0.38*)	7.9 ^ab^	6.6	38.0 ^ab^	10.8 ^abc^	18.0	448.7
SEM	0.247	0.069	0.474	0.142	0.416	18.86
Reference rang *	0–48	5.8–8.1	33–45	10.8–14.8	10–22	220–620
*p*-value						
Treatment ^1^	0.009	0.256	0.002	<0.001	0.197	0.695
Regression Quadratic ^2^	<0.001	0.526	0.020	0.001	0.236	0.839

^1^ *p*-value based on ANOVA (GLM) using treatment and sex as fixed factors (incl. interaction; sex was distributed equally in pens), BW0 was used as a covariate; ^a,b,c^ means with different superscripts differ significantly: *p* < 0.05 (post hoc Tukey HSD); ^2^ *p*-value based on quadratic regression analysis with increasing level of histidine in the diet. * Reference range of healthy individuals given by the laboratory (LABOKLIN GmbH & Co.KG, 97688 Bad Kissingen, Germany). Italic values in brackets refer to the calculated SID His to SID Lysine (Lys) ratio; SID = standardized ileal digestible.

**Table 8:** Carnosine and anserine concentrations in the muscles (*M. longissimus dorsi*) of piglets after 6 weeks of being fed with graded levels of His.

Treatment (*SID His to Lys*)	Carnosine [mg/100 g]	Anserine [mg/100 g]
1 (0.20)	70.0 ^a^	22.3 ^a^
2 (*0.24*)	117.7 ^a^	23.8 ^ab^
3 (*0.27*)	178.2 ^b^	26.8 ^ab^
4 (*0.31*)	251.4 ^c^	25.0 ^ab^
5 (*0.35*)	365.1 ^d^	27.6 ^b^
6 (*0.38*)	383.4 ^d^	24.8 ^ab^
SEM	16.606	0.521
*p*-value ^1^	<0.001	0.028

^1^ *p*-value based on ANOVA (GLM) using treatment as fixed factor; ^a,b,c,d^ means with different superscripts differ significantly: *p* < 0.05 (post hoc Tukey HSD). Italic values in brackets refer to the calculated SID His to SID Lysine (Lys) ratio; SID = standardized ileal digestible.

## Data Availability

The data presented in this study are available on request from the corresponding author.
